# Systematic literature review and narrative synthesis of the effectiveness of health insurance in improving access to healthcare and providing financial protection in Nigeria

**DOI:** 10.3389/frhs.2026.1946888

**Published:** 2026-10-09

**Authors:** Rolle Remi Ahuru, Ngozi Alison Okenya

**Affiliations:** 1 Department of Economics and Development Studies, Federal University Otuoke, Otuoke, Bayelsa State, Nigeria; 2 Department of Economics, Dennis Osadebay University, Asaba, Delta State, Nigeria

**Keywords:** health insurance, healthcare access, financial risk protections, systematic literature, Nigeria

## Abstract

**Background:**

This study conducted a systematic literature review with narrative synthesis to examine the effectiveness of health insurance in improving access to care and reducing out-of-pocket (OOP) health spending among beneficiaries.

**Methods:**

Using PRISMA guidelines, a systematic search was conducted across three databases (Web of Science, PubMed, and Google Scholar). Results were limited to human studies published in English between 2015 and 2024. Studies reporting on the effectiveness of health insurance were included and critically appraised using the Joanna Briggs Institute (JBI) Critical Appraisal Checklist for Analytical Cross-Sectional Studies. The findings were synthesised using a thematic narrative synthesis approach.

**Results:**

The database search identified 495 studies. After removing duplicates and completing title, abstract, and full-text screening, 12 studies were included in the review. From the results, it is suggestive that health insurance has the potential to increase healthcare uptake and reduce excessive dependence on OOP, and hence can guarantee financial protection. However, enrolment remains low and significant disparities exists in enrolment between rural and urban, northern and southern population and educated and non-educated class.

**Conclusion:**

Efforts should be directed towards encouraging enrolment into health insurance, and improve its benefits for enrollees. However, to encourage enrolment, especially among the poor and rural population, Nigerian government should commit itself in full implementation the Vulnerable Group Fund which aims to target over 83 million Nigerians in the informal sector for coverage.

## 1 Introduction

Since the World Health Organization's (WHO) World Health Assembly passed Resolution 58.33 in 2005, there has been increased global focus on the policy challenges associated with health insurance ownership (1, 2). The WHO has consistently encouraged countries to adopt sustainable health financing models that provide financial protection for their citizens (3). Financial protection in healthcare refers to the extent to which individuals or households are protected from the financial burden of paying for health services, particularly when seeking needed care (2). According to Aregbesola and Khan (3), the core objective of universal health coverage (UHC) is to ensure equitable access to quality healthcare while minimizing the risk of financial catastrophe. In Nigeria, the UHC service coverage index stands at a mere 38%, far below the global average of 68% (4).

Health insurance serves as a financial pooling mechanism that spreads the cost of illness across individuals and over time, thereby reducing the financial burden on any single individual (2). As a financing strategy, it protects individuals from high medical costs and plays a crucial role in improving access to affordable and quality healthcare (5–7). In countries such as Peru, Ghana, Indonesia, Vietnam, Colombia, Burkina Faso, China, Mexico, India, and the Lao People's Democratic Republic, health insurance has played significant roles in fostering financial protection, and improving access to care (2). In contrast, the results in Nigeria have been mixed, with several studies reporting catastrophic health expenditures due to a high reliance on out-of-pocket (OOP) health spending (8, 9).

In response to the global call for sustainable health financing models that guarantee financial protection, several African nations have prioritised healthcare reforms and introduced policies to enhance citizens' well-being. The Nigerian government introduced the National Health Insurance Scheme (NHIS) under Decree No. 35 in 1999, representing a major step towards achieving UHC (10). The scheme became fully operational in 2005, with the ambitious goal of achieving UHC for all by 2015 (3). The NHIS functions as a financial pooling mechanism, collecting monthly contributions (premiums) from enrollees to cover the cost of healthcare services as defined in its policy framework (3). Private health insurance exits in Nigeria to cover the wealthy class, though it is underperforming, having provided coverage for less than 1% of the Nigerian population (3). Community-based health insurance programme (CBHIP) function as an arm of NHIS to provide coverage for rural and informal sector, though it is fraught with several challenges (2, 3).

Despite these initiatives, Nigerians continue to face significant challenges in accessing quality healthcare (11). As noted by Primja et al. (12), Nigeria is among the countries with the highest OOP expenditures globally. The country's health financing system remains constrained by endemic corruption, low public awareness, inadequate donor funding, limited insurance coverage, insufficient medical supplies, and low enrolment rates, particularly among individuals in the informal sector (9, 13). In the light of the foregoing, it is pertinent to examine the effectiveness of health insurance in Nigeria as 2030, the terminal year for Sustainable Development Goals (SDGs) draw near. This study is a systematic literature on the effectiveness of health insurance to improve healthcare uptake and reduce OOP health spending.

## 2 Method

This systematic literature review followed the Preferred Reporting Items for Systematic Reviews and Meta-Analyses (PRISMA) 2020 reporting guidelines (14)*.*

### 2.1 Search Strategy

An extensive search was conducted for peer-reviewed studies across multiple electronic databases, including PubMed, Web of Science, and Google Scholar. In addition, the reference lists of all included studies were manually screened to identify relevant articles not captured in the initial search. Google Scholar was included due to its broad coverage of academic materials, particularly grey literature. Unlike more specialised databases, Google Scholar indexes a wide range of sources, making it especially useful for identifying studies that may not be available in traditional databases. The search took place between January 12th, 2026 and February 27th, 2026. The specific search terms employed are presented in Table 1. All duplicate records identified across the databases were carefully reviewed and removed prior to screening. The complete list of search term is provided (Supplementary Additional File 1).

**Table 1:** Search terms.

Search	Query	Items Found (*N*)
PubMed		
S1	(("National Health Insurance Scheme" OR Health Insurance) AND (Nigeria) AND ("healthcare access" OR "access to healthcare" OR "health service utilization") AND ("financial risk protection" OR "financial protection" OR "out-of-pocket" OR "catastrophic health expenditure"))	172
Web of Science (WoS)		
S2	TS= ("National Health Insurance Scheme" OR Health insurance) AND TS=(Nigeria) AND TS= ("healthcare access" OR "access to healthcare" OR "health service utilization") AND TS= ("financial risk protection" OR "financial protection" OR "out-of-pocket" OR "catastrophic health expenditure")	125
Google Scholar		
S3	"Health insurance" Nigeria "healthcare access" "financial risk protection" OR "financial protection" OR "out-of-pocket" OR "catastrophic health expenditure"	198

### 2.2 Screening

All records retrieved from the three databases were exported to and imported into *EndNote* reference management software, where duplicate records were identified and removed. The de-duplicated records were then imported into Covidence, a web-based systematic review platform used for screening articles (5).

Two review authors independently screened the titles and abstracts for eligibility based on the predefined inclusion and exclusion criteria presented in Table 2. Any discrepancies at this stage were resolved through discussion and consensus. Where consensus could not be reached, a third review author was consulted.

**Table 2:** Inclusion and exclusion criteria. The PICO (Population, Intervention, Comparison, and Outcome) framework was used for study selection.

Criteria	Inclusion	Exclusion
Population	- Studies involving individuals, households or specific population groups (e.g., formal sector workers, women or children) - Studies focusing on healthcare users or financial protection linked to health insurance.	- Studies conducted outside Nigeria. - Studies focusing on healthcare usage or financial protection, but not link to health insurance.
Intervention	- Studies explicitly examining health insurance in Nigeria.	- Studies discussing health financing in general, but not link to health insurance.
Comparison	- Healthcare access before and after enrolment into health insurance - Health insurance with other healthcare financing mechanisms in Nigeria.	Not applicable
Outcomes	- Studies relating healthcare access (utilisation, service coverage, equity in access and delay in care). - Studies relating financial risk protections (OOP, catastrophe in health spending and poverty due to healthcare costs).	- Studies focusing exclusively on clinical outcomes such as treatment effectiveness without relationship to financial or access dimensions.
Study design	- Cross-sectional descriptive studies.	- Systematic review/meta- analysis/scoping review^a^. - Studies with inadequate methodological details to support interpretation of results.
Publication characteristics	Studies published in English language, grey literature and studies published between 2015 and 2024.	Commentaries, papers published in non-English, papers without full text retrievals, duplicate studies or earlier version of same analysis and articles published before 2015.

a Systematic reviews and meta-analyses were excluded from this study to make sure only primary empirical studies were included in the review. The focus of this review was to synthesis original data and generate context-specific insights that are relevant to the research objectives. Including secondary studies such as systematic reviews could lead to duplicate of evidence, as these studies often aggregate findings from primary research that may have been captured in the review.

Full-text articles of studies deemed potentially eligible were retrieved and independently assessed by two review authors for final inclusion. Discrepancies at the full-text screening stage were resolved through discussion, with a third reviewer consulted when necessary to reach consensus.

## 3 Eligibility criteria

### 3.1 Quality assessment and risk of bias

To assess the quality of the included papers, the Joanna Briggs Institute (JBI) Critical Appraisal Checklist for Analytical Cross-Sectional Studies was employed to evaluate methodological quality (15). The tool consists of eight criteria covering five domains: selection and sampling, exposure measurement, outcome measurement, identification and management of confounding factors, and appropriateness of statistical analysis (16). Each criterion was rated using four possible responses: Yes (score = 1), No (score = 0), Unclear, and Not applicable (17). Based on the total scores obtained, studies were categorised as good quality (6–8 points), fair quality (4-5 points), or poor quality (0–3 points) (17).

Two review authors independently conducted the quality assessment using the predefined appraisal criteria. Discrepancies were resolved through discussion and consensus. Where consensus could not be reached, a third person was consulted to make the final decision. This process ensured that only studies meeting an acceptable quality threshold were included in the synthesis.

Grey literature was included to reduce publication bias and ensure comprehensive coverage of available evidence. It was searched using Google Scholar with the same search terms applied to peer-reviewed studies. These sources were appraised using JBI critical appraisal tools. While grey literature provided additional insights, variations in reporting quality and the absence of peer review were taken into account when interpreting the findings.

### 3.2 Data extraction

The data extraction form was piloted, tested, and revised in Microsoft Excel. Data were extracted by one reviewer and verified by a second reviewer. Relevant information was collected using a standardised data extraction form, capturing details such as author(s), year of publication, study location/setting, study design, sample characteristics, and key findings. Studies that lacked definitive results or presented only recommendations without empirical evidence were excluded.

### 3.3 Data synthesis

Due to the differences in the outcome measures (e.g., healthcare utilisation, financial protection and enrolment equity), a quantitative meta-analysis was not carried out. A structured thematic synthesis approach was employed to guide the narrative synthesis. Extracted findings were reviewed iteratively, coded according to recurring concepts, and subsequently organized into descriptive and analytical themes. In Table 3, the themes and the subthemes are presented. Potential biases and methodological limitations were considered during interpretation, with greater emphasis placed on studies with more robust methodologies. A summary of the findings of the included studies is provided on the online Supplementary Additional File 2.

**Table 3:** Themes and sub-themes identified from reviewed studies.

Themes	Subthemes	References
Impact of health insurance on service accessibility	Utilisation of care	(5, 18–26)
	Enrolment rate/Inequity	(5, 23, 24, 27, 28)
Level of financial protection by insurance coverage	Effects on out-of-pocket spending	(20, 22, 23, 25, 26)

### 3.4 Search outcomes

A total of 495 studies were initially identified through searches across three databases using the specified search terms. Of these, 307 studies were removed based on title screening and duplication. After screening the articles by their abstract, 122 were found not to meet the inclusion criteria. The remaining 66 full-texts were assessed for eligibility. Out of these 54 papers were excluded because their focus didn't align with the subject matter**.** Consequently, 12 studies that met the quality assessment criteria were included in the review. The study selection flowchart is presented in Figure 1.

**Figure 1:**
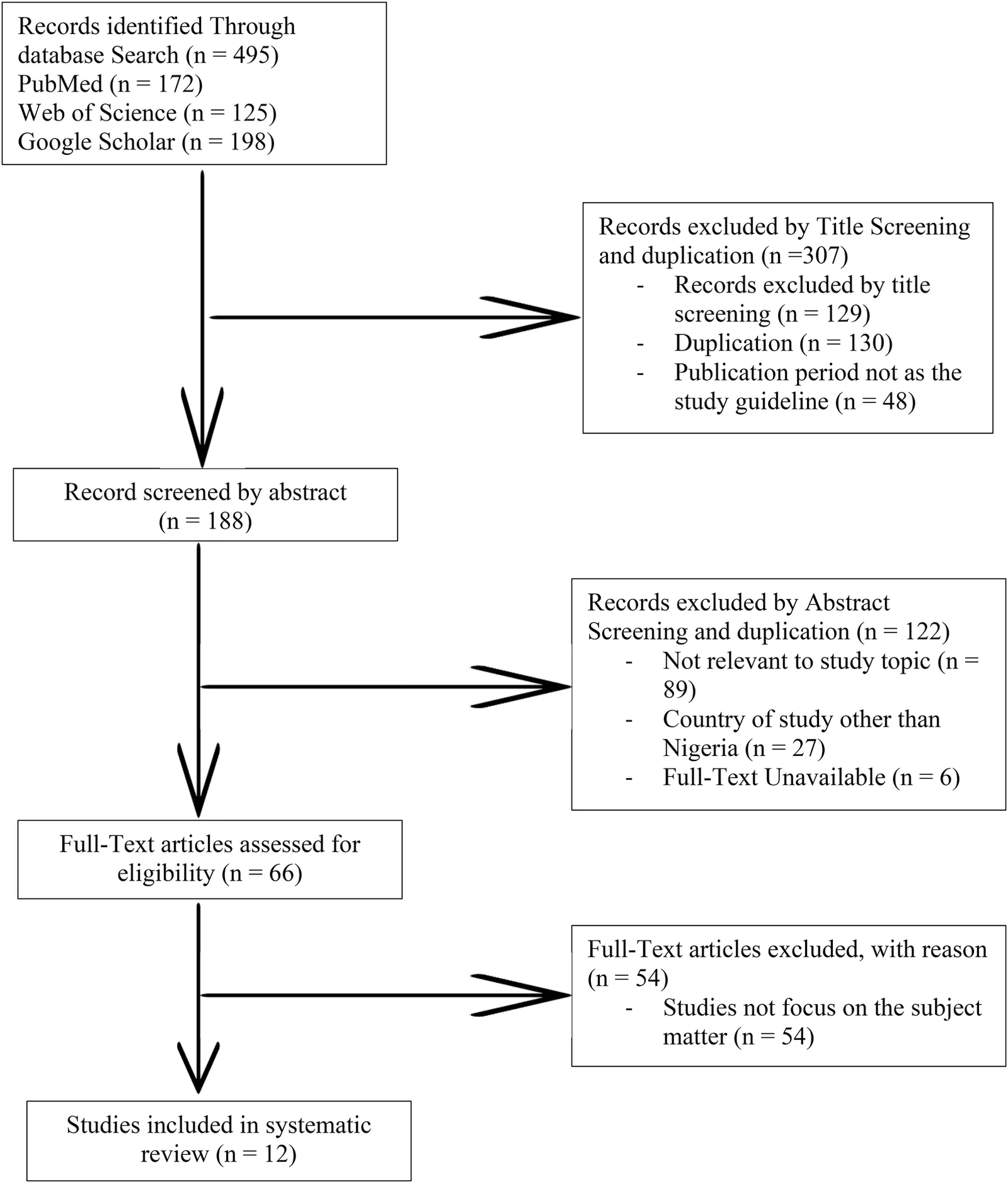
PRISMA Flow chart**.**

### 3.5 Assessment of methodological quality

All the articles included in this study were rated as good, having scored 6–8 out of the 8 assessment questions. The twelve (12) studies included in the review were found to be satisfactory using the quality assessment tool. A detailed explanation of the quality assessment procedures and results is given in the Online Supplementary Additional file 3.

### 3.6 Limitations of the reviewed studies

The reviewed studies exhibit several methodological limitations that may affect the validity, reliability, and generalisability of their findings. These limitations span selection bias, measurement bias, recall bias, and confounding.

A key limitation common across the studies is confounding bias. Several studies e.g., 5, 26, which utilised cross-sectional DHS data, examined the association between national health insurance and maternal healthcare utilisation at a single point in time. Although logistic regression and multilevel models were employed to control for observable characteristics, there remains a risk of residual confounding. In particular, these studies did not adequately account for unmeasured contextual factors such as cultural beliefs, quality and availability of healthcare facilities, distance to facilities, and health system capacity. The omission of these variables may have influenced the observed relationship between insurance enrolment and healthcare access.

Selection bias is another important limitation evident across the reviewed literature. Studies that collected primary data often focused on urban residents or individuals who had already accessed healthcare services (18–21, 25–28). This sampling approach is likely to overrepresent individuals who are more frequent users of health services, thereby limiting the generalisability of findings to urban populations and more accessible or “overserved” groups. In addition, large-scale surveys such as the DHS, as well as reports like those from Nigerian Health Watch (22), may underrepresent highly marginalised populations, including nomadic groups, individuals in conflict-affected areas, and extremely low-income households who are less likely to be captured in surveys or to access health insurance. This underrepresentation may lead to biased estimates of both insurance enrolment and healthcare utilisation. Furthermore, grey literatures such as DHS outcomes tend to provide descriptive distributions without fully addressing structural inequalities that may explain variations in health insurance enrolment across wealth, geography, and other socio-demographic factors.

Recall bias also represents a recurring limitation across the studies and technical reports. Respondents were often required to report past healthcare experiences, expenditures, or insurance-related information over extended periods. This reliance on memory may result in inaccurate reporting due to imperfect recall. Such bias may lead to either overestimation or underestimation of healthcare utilisation and expenditure patterns. Additionally, Iloh et al. (18), who employed a four-point Likert scale, may also be subject to response reconstruction, where participants answer based on current perceptions rather than actual past experiences. This can distort findings regarding the impact of insurance on financial protection and healthcare utilisation.

Measurement bias is also evident in several studies due to the operationalisation of key variables. For example, Ahuru et al. (5) and Zhang et al. (24) relied on simple binary (yes/no) measures to capture health insurance enrolment. This approach oversimplifies a complex construct and may introduce social desirability bias, as respondents could provide answers, they perceive as socially acceptable. Furthermore, studies such as Ahuru et al. (5), using DHS data, classified individuals solely as enrolled or not enrolled without considering important dimensions such as duration of coverage, benefit package differences, or continuity of enrolment. These distinctions are important, as different insurance schemes may offer varying levels of financial protection and access to healthcare services.

### 3.7 Characteristics of included papers

The descriptive statistics of the included studies is presented in Table 4. It can be seen that a total of 12 papers were used for the analyses. All the 12 papers employed a quantitative study design. Most of the studies were published between 2019 and 2021 (50.0%, *n* = 6), next 2022-2024 (25.0%, *n* = 3), and 2015-2018 (25.0%, *n* = 3). Concerning data collection methods, 15.4% (*n* = 2) used secondary data, and 84.6% (*n* = 11) used primary data. Also, 16.7% (*n* = 2) used secondary data from Demographic and Health Survey, 83.3% (*n* = 10) used questionnaire in collecting data.

**Table 4:** Characteristics of included studies (*n* = 12).

General characteristics of the studies	*N*(%)	Study references
Study method:		
Qualitative	-	-
Quantitative	12 (100.0)	(5, 18–22, 24–28)
Study year:		
2015-2018	3 (25.0)	(18, 23, 25).
2019-2021	6 (50.0)	(5, 19, 20, 26–28)
2022-2024	3 (25.0)	(21, 22, 24)
Data source:		
Primary	10 (83.3)	(18–23, 25–28)
Secondary	2 (16.7)	(5, 24)
Method of data collection:		
Demographic and health survey	2 (16.7)	(5, 24)
Primary questionnaire	10 (83.3)	(18–23, 25–28)

## 4 Results

### 4.1 Impact of health insurance on service accessibility

#### 4.1.1 Utilisation of care

Ten studies (5, 14, 15, 16, 19, 24, 25, 26, 27, 28) reported on the impact of health insurance on healthcare uptake and willingness to use healthcare services. Two studies used secondary data from the DHS (23) to evaluate the impact of enrolment into health insurance on maternal healthcare utilization see 5, 26. Ahuru et al. (5) analysed a nationally representative sample of 8,006 women of reproductive age using binary logistic regression and found a statistically significant positive association between enrolment into health insurance and the utilization of various maternal healthcare services, including antenatal care (ANC), skilled birth attendants (SBA), and postnatal care (PNC). Similarly, Zhang et al. (24), using a representative sample of 6,761 women, found that women with health insurance coverage were more likely to deliver in health facilities than those who were not enrolled. These studies provide relatively robust evidence because they drew on nationally representative datasets and employed multivariable regression techniques that adjusted for potential confounding factors. The consistency of their findings strengthens the evidence that enrolment into health insurance is associated with increased uptake of maternal and reproductive healthcare services. Nevertheless, the cross-sectional nature of the data limits causal inference, as reverse causality and residual confounding may still be present.

The NDHS (23), based on a nationally representative household survey comprising 41,668 households, 42,121 women, and 13,311 men, similarly reported higher uptake of various maternal healthcare services among women covered by health insurance, including ANC, PNC, and child immunization. Although these descriptive findings align with those of Ahuru et al. (5) and Zhang et al. (24), the NDHS analysis relied primarily on frequencies and proportions and therefore could not account for the influence of confounding factors. Nevertheless, the convergence of findings across both descriptive and multivariable analyses suggests a stable positive relationship between enrolment into health insurance and maternal healthcare utilization.

Evidence from smaller facility-based studies generally supports these national-level findings. Adedinsewo et al. (19), in a cross-sectional study among NHIS enrollees attending accredited facilities in urban areas of Osun State, found that immunization coverage was higher among children whose mothers were enrolled in NHIS, and that NHIS enrollees reported a greater willingness to seek care from healthcare professionals. Similarly, Olusola et al. (21) reported higher healthcare utilization and lower OOP health expenditure among NHIS enrollees in accredited hospitals in Ogun State, while Okunade et al. (20) found that NHIS enrollees reported greater uptake of preventive healthcare services in accredited facilities in Lagos State. The consistency in the direction of findings across these studies suggests that NHIS enrolment promotes positive healthcare-seeking behaviour and encourages access to both preventive and curative healthcare services.

However, these smaller studies provide less robust evidence than the NDHS-based studies because they relied on facility-based samples that were largely drawn from urban areas and predominantly employed descriptive analytical methods. Their respondents were already users of NHIS-accredited facilities and therefore may have had better access to health insurance and healthcare services than the general population. Consequently, the findings may overestimate the benefits of insurance uptake compared with nationally representative studies that include both users and non-users of healthcare facilities.

Overall, the strongest evidence from nationally representative NDHS-based studies demonstrates a positive association between insurance uptake and healthcare utilization among women. Findings from smaller facility-based studies largely reinforce this conclusion, although their limited geographical coverage, urban focus, and reliance on descriptive analyses warrant cautious interpretation. Taken together, the evidence suggests that insurance uptake is positively associated with the utilization of maternal healthcare, preventive healthcare, and general healthcare services.

#### 4.1.2 Enrolment rates/inequity in access

Five of the reviewed studies (5, 23, 24, 27, 28) reported on insurance enrolment rates. Across these studies, a consistent pattern emerged, indicating generally low insurance uptake, particularly among vulnerable populations and individuals working in the informal sector. The studies also revealed marked socioeconomic and demographic inequalities in enrolment, especially with respect to wealth status, place of residence, educational attainment, and geographical region.

The most robust evidence was derived from nationally representative analyses of the NDHS (23), as well as secondary analyses of the same dataset by Ahuru et al. (5) and Zhang et al. (24). These studies, which benefited from large sample sizes and, in most cases, employed multivariable analytical techniques, consistently demonstrated higher enrolment among urban residents, wealthier households, individuals with higher educational attainment, and women residing in Southern Nigeria. According to the NDHS (23), the insurance coverage rate among urban residents (23.9%) was approximately twice that of rural residents (11.8%), highlighting substantial spatial inequalities in insurance uptake.

Evidence from smaller, state-level studies provided complementary but less robust support for these patterns. For instance, Oladosu et al. (27) reported marked urban-rural disparities in NHIS enrolment in Osun State, while Osinowo et al. (28) similarly documented disparities in enrolment and health system performance between facilities located in rural and urban areas of the state. Although these studies were limited by smaller sample sizes and largely descriptive analytical approaches, their findings closely align with those from national household surveys. This suggests that the observed inequalities are not merely artefacts of national-level aggregation but are also evident at the state level.

Collectively, these findings indicate that insurance uptake in Nigeria remains low and structurally unequal, with enrolment concentrated among socioeconomically advantaged and urban populations. This pattern suggests that the benefits of insurance enrolments are unevenly distributed, as individuals in rural communities and the informal sector continue to face persistent barriers to enrolment. These barriers may include limited access to accredited healthcare facilities, difficulties navigating registration procedures, inadequate awareness of insurance benefits, and poor knowledge of the availability of the schemes.

While the convergence of findings across multiple datasets and study designs strengthens confidence in the observed socioeconomic inequalities, it is important to note that most of the studies employed cross-sectional designs and relied primarily on descriptive analyses. Consequently, they provide limited insight into the causal mechanisms driving enrolment inequalities, including gaps in policy implementation, supply-side constraints, and behavioural determinants of health insurance uptake.

### 4.2 Level of financial risk protection by health insurance

#### 4.2.1 Effects on out-of-pocket spending

Five studies (20, 22, 23, 25, 26) consistently demonstrated that health insurance enrolment is associated with lower OOP health expenditure and improved financial protection among beneficiaries. However, the strength of this evidence varies considerably according to study design and representativeness, with nationally representative surveys providing more robust estimates than smaller facility-based studies.

The strongest evidence comes from the NDHS (23) and the Nigerian Health Watch (22) report. Both studies employed multi-stage sampling techniques and included insured and uninsured populations across multiple regions, thereby allowing more reliable comparisons of financial outcomes. The NDHS (23) specifically reported that women enrolled in NHIS incurred substantially lower OOP expenditure for maternal and reproductive healthcare services than uninsured women, suggesting that financial protection is particularly important in high-cost areas of care.

Similarly, Nigerian Health Watch (22) reported clear differences in average annual per capita OOP expenditure, with insured individuals spending approximately ₦11,142 compared to ₦24,345 among the uninsured. This difference suggests meaningful financial savings associated with insurance coverage and suggestive of the facts that health insurance coverage may result in the reduction of the risk of catastrophic health expenditure. Because these findings were derived from large-scale household survey data, they provide relatively strong evidence of population-level financial protection effects.

In contrast, facility-based studies such as Akinwale et al. (25) and Odeyemi et al. (26), conducted in Ondo State and Ogun State, respectively, also reported reduced financial burdens among NHIS enrollees, but within more restricted and potentially biased samples. Odeyemi et al. (26) found that enrollees were less likely to experience financial hardship than non-enrollees, while Akinwale et al. (25) reported that uninsured households devoted a larger proportion of their income to healthcare and relied heavily on direct OOP payments. However, the generalisability of these findings is limited by the use of relatively small, facility-based samples and self-reported expenditure data, which may overrepresent healthcare users and introduce recall bias.

Okunade et al. (20), in a study conducted in Lagos State, further supported this pattern by reporting a reduced financial burden among insurance beneficiaries. However, the study primarily employed descriptive analytical methods and was limited to NHIS-accredited facilities located in urban areas. Consequently, its findings are context-specific and less generalisable to rural populations or individuals who are not enrolled in NHIS.

Across all studies, a common methodological feature is the reliance on self-reported expenditure and perceived financial hardship measures. While this facilitates comparison across studies, it also introduces the possibility of recall and reporting bias, which may result in the underestimation or overestimation of actual OOP expenditure. This limitation is particularly relevant in facility-based studies, where respondents are already engaged with the healthcare system.

Overall, the evidence converges on a clear directional conclusion: insurance uptake is associated with lower OOP expenditure and improved financial protection. However, the strength of the evidence is not uniform. Nationally representative household surveys, such as the NDHS (23) and Nigerian Health Watch (22), provide stronger and more generalisable support for this conclusion, while smaller cross-sectional facility-based studies largely confirm the same trend within more limited and context-specific populations.

## 5 Discussion of the results

This systematic literature review adopted a narrative synthesis approach to integrate findings from diverse sources on the impact of health insurance uptake on healthcare accessibility and its effectiveness in providing financial protections. A total of twelve (12) peer-reviewed studies were synthesised. Unlike previous studies, this review employed a narrative synthesis to assess the role of health insurance uptake in fostering healthcare utilisation and safeguarding individuals against financial hardship in funding healthcare.

From the reviewed papers see 5,25, 26, it is seen that uptake of health insurance remains abysmally low. This aligns to the report made by similar studies See 13, 29-30. The low uptake of health insurance is caused by several factors, prominent among which are low level of awareness for the existence of the scheme, high proportion of Nigerians are engaged in the informal sector (> 90%), lack of trust, and lack of money to pay as premium (29). The result is similar to the findings across Africa, with only four African countries having ≥ 20% of their population covered by health insurance: Rwanda, 79%; Ghana, 58%; Gabon, 41%, and Burundi, 22%, of the 36 countries in sub-Saharan Africa assessed in 2022 (30). Countries that have ≥ 20% of their population covered by the social health insurance have broadened the scope of CBHIP into the informal sector, subsidise insurance premium for the poor, and vulnerable sections of the society. For instance, Rwanda has covered a large proportion of its population through CBHIP, and Ghana has opted to fund its social health insurance through taxes and contributions, hence reducing the burden on households who intend to get covered. Therefore, Nigeria government should commit itself to the full implementation of the Vulnerable Group Fund (VGF) under the NHIA act, 2022 which aims to target 83 million vulnerable and indigent Nigerians which is about 38% of the population (13).

Five of the studies revealed that insurance uptake is associated with reduction in OOP health spending, and as a result, enrolment into health insurance may be associated with a reduction in OOP (20, 22, 23, 25, 26). Some of the studies reported that following enrolment into social health insurance, OOP health spending significantly reduced (25, 26). Similar findings have been made for other African countries such as Ghana (33) and South Africa (31), where enrolment into social insurance programme reduces OOP health spending. Based on the reviewed papers, insurance uptake may have the potentials to provide financial safety net, guarantee financial protections, and empower the poor and vulnerable sections of the society to use modern healthcare services (32). It, therefore, follows that broadening the scope of social health insurance programme and scaling up CBHIP will help the country in achieving the SDGs 3.8, which targets financial protections in healthcare usage.

The majority of the reviewed studies indicate that health insurance uptake is associated with increased healthcare utilisation. Evidence consistently shows that individuals covered by health insurance were more likely to utilise healthcare services than their counterparts not covered (20, 27). For instance, Ahuru et al. (5) and Zhang et al. (24) found that insurance uptake is correlated with the utilisation of maternal healthcare services, including ANC, SBA, and PNC. Similar findings have been reported in other Nigerian studies (26, 29) and in studies conducted in other countries (33, 34). However, important disparities in enrolment persist, disproportionately favouring educated individuals, wealthier households, urban residents, and populations in Southern Nigerian (21–23, 25).

These findings can be critically interpreted using the Andersen's Healthcare Utilisation Model, which conceptualises healthcare utilisation as a function of predisposing, enabling, and need-based factors operating within broader social and health system contexts (35). The observed inequalities in insurance uptake demonstrate that healthcare utilisation is not determined solely by individual health needs, but is deeply shaped by social stratification and structural inequalities. Within Andersen's framework, predisposing factors refer to socio-demographic characteristics that influence an individual's propensity to use healthcare services (36). The concentration of insurance enrolment on the educated, the wealthy, urban and Southern populations suggest that social location significantly shapes health-seeking behaviour and insurance participation (5). Education, in particular, emerged as a major determinant of enrolment across the reviewed studies (21–23, 25). Higher levels of education enhance health literacy, improve awareness of insurance benefits, strengthen ability to navigate registration procedures and increase trust in formal healthcare systems (13). Educated individuals are, therefore, more likely to understand the principles of risk pooling and recognise the long-term financial benefits of health insurance participation (29). In contrast, uneducated persons may perceive premium payments as immediate financial burdens rather than future financial protection, especially within contexts of widespread economic insecurity (13). This suggests that inequalities in education indirectly translate into healthcare inequalities through differential access to insurance enrolment.

Geographical disparities in insurance uptake further illustrate the role of predisposing factors in shaping healthcare utilisation. The reviewed studies consistently show that residents of Southern Nigeria are more likely to be covered by health insurance than their Northern counterparts (21–23, 25). This pattern reflects longstanding historical, political, educational, and socioeconomic inequalities between the two regions (37). Southern Nigeria generally records higher literacy rates, greater formal-sector employment, and better healthcare infrastructure than Northern Nigeria (38). Consequently, higher enrolment in the South cannot be understood simply as a matter of individual preference; rather, it reflects unequal structural opportunities that condition access to healthcare and insurance participation. This interpretation aligns with Andersen's argument that healthcare utilisation is socially patterned and embedded within wider structural determinants.

The reviewed studies also reveal persistent urban-rural disparities in NHIS enrolment (13, 32). Urban residents are more likely to be exposed to health insurance awareness campaigns, formal employment opportunities linked to insurance coverage, and insurance-accredited healthcare facilities (13). In contrast, rural populations are disproportionately concentrated in informal economic activities and often rely on informal or traditional healthcare providers, thereby limiting both insurance enrolment and formal healthcare utilisation (39). Moreover, Nigeria's historically employment-based insurance structure has largely favoured formal-sector workers while excluding individuals in informal employment and unpaid labour (23). This indicates that the design of the social health insurance itself may unintentionally reproduce existing social inequalities by privileging populations already advantaged within the labour market.

Beyond predisposing characteristics, Andersen's model emphasises the importance of enabling factors, which refer to the practical resources that facilitate healthcare utilisation. Predisposing factors include income, insurance coverage, service availability, and institutional arrangements (35). The reviewed evidence suggests that enrolment into health insurance functions as a critical enabling resource, hence is associated with a reduction in OOP, and increased healthcare utilisation (20, 22, 23, 25, 26). However, the unequal distribution of enabling resources undermines the equity objectives of the scheme. Wealthier households possess greater financial capacity to pay premiums, cover transportation costs, and absorb the indirect costs associated with seeking healthcare (5). As a result, populations already experiencing socioeconomic advantage are more capable of benefiting from insurance coverage (32).

This finding reflects broader critiques within health systems literature regarding insurance-based financing mechanisms in low-and middle-income countries. In contexts characterised by large informal sectors and widespread poverty, formal health insurance schemes may inadvertently reinforce existing inequalities rather than function as universal protection mechanisms (5, 13). Instead of promoting equitable access, such systems may become segmented, disproportionately benefiting formally employed and economically advantaged groups while marginalising vulnerable populations. The findings also align with the Social Determinants of Health framework, which emphasises that healthcare utilisation and insurance participation are shaped by broader socioeconomic and political conditions rather than by individual choice alone 5; 39. Factors such as poverty, educational inequality, regional underdevelopment, gender disparities, and labour market structures significantly influence access to health insurance and healthcare services. Consequently, inequalities in insurance uptake should not be viewed merely as policy implementation gaps, but as manifestations of deeper structural inequalities within Nigerian society (39).

From a UHC perspective, these findings suggest that expanding nominal insurance coverage alone is insufficient to achieve equitable healthcare access. Genuine universality requires equitable financing mechanisms, improved geographical distribution of healthcare facilities, inclusion of informal-sector workers, gender-sensitive policy frameworks, the reduction of non-financial barriers to healthcare access and implementing tax-based non-contributory scheme among people in the informal sector (40). Policy efforts should, therefore, extend beyond increasing enrolment figures to addressing the structural determinants that limit participation among vulnerable populations. This includes targeted subsidies, premium waivers, and exemption policy for rural populations, unemployed individuals, and low-income households. The establishment of the VGF under the NHIA act represents an important policy step towards improving inclusion among disadvantaged populations since it is difficult to collect premium from the poor and those in the informal sector (13). However, its effectiveness will ultimately depend on sustained political commitment, adequate funding, increasing service coverage especially in the rural parts of the country, reducing population growth rate, strengthen accountability and governance structure.

## 6 Limitations

Despite its strengths, the study has several noteworthy limitations. First, many of the studies included in the review adopted quantitative research methods. While quantitative approaches are valuable for measuring outcomes, the structured nature of questionnaires used for data collection may restrict respondents' ability to fully express their subjective and personalized experiences. Consequently, important contextual insights and experiences of those enrolled into health insurance may not be adequately captured.

The second limitation is the heterogeneity in the outcome measures of the included studies. Although the majority of the studies employed quantitative methods, due to differences in outcome measures, meta-analyses couldn't be conducted, and this limits the ability to quantify pooled effects and may reduce the precision of conclusions.

Third, the majority of the studies included in the review focused on South-Western region, and this geographical clustering limits national generalizability. However, this was irresistibly the case because the majority of the studies were conducted in that region.

## 7 Conclusion and recommendations

This study synthesises evidence on the impact of insurance uptake on healthcare access and financial protection. The review suggests that insurance uptake may be associated with healthcare uptake and as a result, may lessen the excessive dependence on OOP health spending. However, overall enrolment remains low and highly inequitable, disproportionately favouring urban, southern, wealthier, and more educated populations. It is suggestive that effectiveness of the scheme is further constrained by operational inefficiencies, including long waiting times, weak patient-provider interaction, poor diagnostic practices, and frequent unavailability of prescribed medicines. To address these challenges, the study recommends commitment in implementing the VGF under NHIA act, increase service coverage especially strengthening Primary Healthcare Centers, boosting government expenditure on health through broadening tax revenue and scaling up CBHIP in the informal sector.

## Data Availability

The original contributions presented in the study are included in the article/Supplementary Material, further inquiries can be directed to the corresponding author.
