# Supplementary material for: Systematic literature review and narrative synthesis of the effectiveness of health insurance in improving access to healthcare and providing financial protection in Nigeria
Source: Front Health Serv. 2026 Oct 9;6:1946888. doi: 10.3389/frhs.2026.1946888 (PMC13690934; doi:10.3389/frhs.2026.1946888)
Supplement: Supplementary file 1 [file Supplementaryfile1.docx]

**Supplementary Additional File 1: Search Terms (With Filters)**

| **Search** | **Query** | **Items Found (N)** |
| --- | --- | --- |
| **PubMed** | | |
| **S1** | (("National Health Insurance Scheme"[tiab] OR NHIS [tiab]) AND (Nigeria [Mesh] OR Nigeria[tiab]) AND ("Health Services Accessibility"[Mesh] OR "healthcare access"[tiab] OR "access to healthcare"[tiab] OR "health service utilization"[tiab]) AND ("Financial Protection"[tiab] OR "Insurance, Health"[Mesh] OR "out-of-pocket"[tiab] OR "catastrophic health expenditure"[tiab])) AND ("2015/01/01"[Date - Publication]: "2024/12/31"[Date - Publication]) AND Humans [Mesh] AND English[lang] | 172 |
| **Web of Science (WBS)** | | |
| **S2** | TS=("National Health Insurance Scheme" OR NHIS)AND TS=(Nigeria) AND TS=("healthcare access" OR "access to healthcare" OR "health service utilization" OR "healthcare utilization") AND TS=("financial risk protection" OR "financial protection" OR "out-of-pocket" OR "catastrophic health expenditure" OR "out of pocket expenditure") AND PY=(2015-2025) AND DT=(Article OR Review) AND LA=(English) AND WC=("Public, Environmental & Occupational Health" OR "Health Policy & Services" OR "Health Care Sciences & Services"). | 125 |
|  | **Google Scholar** |  |
| **S3** | "National Health Insurance Scheme" Nigeria "healthcare access" "financial risk protection" OR "financial protection" OR "out-of-pocket" OR "catastrophic health expenditure" | 198 |
