# Supplementary material for: Systematic literature review and narrative synthesis of the effectiveness of health insurance in improving access to healthcare and providing financial protection in Nigeria
Source: Front Health Serv. 2026 Oct 9;6:1946888. doi: 10.3389/frhs.2026.1946888 (PMC13690934; doi:10.3389/frhs.2026.1946888)
Supplement: Supplementary file 2 [file Supplementaryfile2.docx]

**Supplementary Additional File 2: Summary of Findings**

| **Author (s)** | **Study location/Setting** | **Study designs** | **Findings** |
| --- | --- | --- | --- |
| Ahuru et al [5] | Nigeria | Cross-sectional Analytical | Low enrolment into NHIS, disparity in enrolment, favouring the urban, wealthy, educated, employed and Southern Nigerian women. NHIS enrolment improves access to maternal care services, especially antenatal care and delivery care. |
| Zhang et al [24] | Nigeria | Cross-sectional Analytical | Enrolment into NHIS enrolment in particular is low, but into health insurance as a whole is also low.  Disparity exists in NHIS enrolment favouring the wealthy, educated, urban and employed women.  A higher share of women who enrolled into NHIS had facility delivery, when compared to those who didn’t enrol. While 34.6% of those enrolled into NHIS delivered in health facilities, and 16.2% of those who didn’t enrol deliver in health facilities. |
| Demographic & Health Survey [25] | Nigeria | Cross-sectional  Descriptive | Low enrolment into NHIS in particular and health insurance in general (< 3.0%).  Inequity in enrolment into health insurance favouring educated, wealthy, urban and southern regions.  Facilities in rural parts of the country lack resources compared to those in urban parts. This unequal ownership of resources affects NHIS-deployed facilities in rural parts of the country. |
| Nigeria Health Watch [22] | Nigeria | Cross-sectional  Descriptive. | Concentration of health facilities in urban parts of the country, while several communities in rural parts of the country don’t have access to health cares due to lack of facilities.  NHIS-deployed facilities often lack adequate storage of drugs, and as a result, many enrollees weren’t administered with prescribed drugs due to unavailability of the recommended drugs.  Average annual per capita Out-of-pocket health spending for those who enrolled was N11,142 ($26) and those who didn’t enrol was N24,345 ($56). |
| Adedinsewo et al [19] | South-Western Nigeria  (Osun State) | Cross-sectional Descriptive | NHIS enrolment increased willingness to seek and improved access to professional care services.  NHIS increased child immunisation rates. Child immunisation rates were higher among mothers covered by NHIS, compared to those not covered. |
| Olusola et al [21] | South-Western Nigeria  (Ogun State) | Cross-sectional Descriptive | NHIS enrolment improves willingness to seek medicare. Also, access to preventive cares services and frequency of visits to health facilities improved following enrolment. Enrolment into NHIS reduced OOP from N782.2 ($4.89) per month to N304.4 ($19.0) prior to coverage. However, access to advanced diagnostic care remains low, and waiting times to see healthcare professionals. |
| Okunade et al [20] | South-Western Nigeria (Lagos State) | Cross-sectional  Descriptive. | Enrolment into NHIS improves access to health services. For instance, there was a 17.2% increase in healthcare utilisation post-NHIS, particularly preventive care in PHCs**.** Ability to access health facilities improved due to the reduction of payments, and sensitisation created by enrolment. |
| Oladosu et al [27] | South-Western Nigeria (Osun State). | Cross-Sectional  Descriptive | The study found out that a higher proportion of respondents from urban parts of the country was enrolled into NHIS, compared to respondents from rural parts of the country.  Also, the study reported disparity in health resources between rural and urban health facilities. For instance, facilities in urban parts of the country had higher hospital bed density compared to facilities in rural parts of the country. |
| Ganokroj et al [28] | South-Western Nigeria (Osun State) | Cross-sectional  Descriptive | Disparity exists in NHIS enrolment between rural and urban residents. While 12% of urban residents enrolled, only 2% of rural residents enrolled into NHIS.  Furthermore, the study reported poor interactions between NHIS-enrollees and healthcare professionals. The feedback from healthcare professionals to patients under NHIS was poor. This result is based on a yes or no response. |
| Iloh et al [18] | South-Western  (Osun State) | Cross-sectional Descriptive | NHIS enrolment has significantly and positively influenced the willingness to seek prompt medical help and utilise preventive health services. Using the four-point Likert scale, 47.5% agreed that they experience reduction in OOP following enrolment into NHIS. Also, 43.8% concurred that enrolment encouraged ability to visit health facilities frequently, and 48.8% concurred that enrolment aids their ability to seek preventive healthcare services (e.g., regular check-ups and screening), 51.8% reported that NHIS improves their willingness to seek medical help. Furthermore, 52% of the respondents concurred that NHIS enrolment reduced the waiting time for scheduling medical appointments, and 46.2% concurred that they still face financial barriers in accessing health services, meaning that NHIS hasn’t redress financial barriers.  Poor working conditions influence service provisions in rural parts of the country. The study reported high rate of healthcare workers’ attrition from rural parts of the country to urban parts, affecting access to modern care services among NHIS enrollees in rural parts of the country. |
| Odeyemi [26] | South-Western  Nigeria  (Ogun State) | Cross-sectional  Descriptive. | According to the results, 88.1% reported improved access to health facility following enrolment into NHIS, 93.9% were able to access clinic anytime due to coverage. The assessment of client satisfaction showed that 73.7% of respondents were satisfied with the quality of care from NHIS. While those who enrolled into NHIS spends up to 20% of their monthly earnings on health, for those who didn’t enrol, their monthly health rising is up to 40%, meaning they were exposed to catastrophe in health spending. |
| Akinwale et al [25] | South-Western Nigeria  (Ondo State) | Cross-sectional Descriptive. | NHIS increases access to modern medical cares and reduced the burden from using modern care services. The study compared OOP health spending for those who enrolled into health insurance with those who didn’t. The results showed that OOP is higher among those who didn’t enrol compared to those who enrolled. Those who enrolled into NHIS spends less than 30% of their total income as medical expenses, while those who didn’t enrol spend up to 40%, meaning incidence of catastrophe. Unsatisfaction with NHIS was associated with poor interactions with healthcare providers. Those who enrolled complained of dismissive attitude and hurried diagnoses. |
