# Supplementary material for: Systematic literature review and narrative synthesis of the effectiveness of health insurance in improving access to healthcare and providing financial protection in Nigeria
Source: Front Health Serv. 2026 Oct 9;6:1946888. doi: 10.3389/frhs.2026.1946888 (PMC13690934; doi:10.3389/frhs.2026.1946888)
Supplement: Supplementary file 3 [file Supplementaryfile3.docx]

**Supplementary Additional File 3: Quality Assessment Tools for Included Studies**

| **S/N** | **Author (s)** | **1** | **2** | **3** | **4** | **5** | **6** | **7** | **8** | **Total**  **Score** | **Quality of study** |
| --- | --- | --- | --- | --- | --- | --- | --- | --- | --- | --- | --- |
| 1 | Adedinsewo et al[19] |  |  |  |  |  |  |  |  | 6 | Good |
| 2 | Nigerian Health Watch [22] |  |  |  |  |  |  |  |  | 7 | Good |
| 3 | Olusola et al [21] |  |  |  |  |  |  |  |  | 6 | Good |
| 4 | Okunade et al [20] |  |  |  |  |  |  |  |  | 6 | Good |
| 5 | **N**igerian Demograaphic & Health Survey [23] |  |  |  |  |  |  |  |  | 7 | Good |
| 6 | Oladosu et al [27] |  |  |  |  |  |  |  |  | 6 | Good |
| 7 | Ganokroj et al [28] |  |  |  |  |  |  |  |  | 6 | Good |
| 8 | Iloh et al [18] |  |  |  |  |  |  |  |  | 6 | Good |
| 9 | Akinwale et al [25] |  |  |  |  |  |  |  |  | 6 | Good |
| 10 | Odeyemi [26] |  |  |  |  |  |  |  |  | 6 | Good |
| 11 | Ahuru et al [5] |  |  |  |  |  |  |  |  | 8 | Good |
| 12 | Zhang et al [24] |  |  |  |  |  |  |  |  | 8 | Good |

**Quality assessment of quantitative studies (n= 13)**

Quality was rated as poor (0-3 score out of 8), rated as fair (4-5 score out of 8) and rated as good (6-8 out of 8).

| Category | Yes | No | NC/NA |
| --- | --- | --- | --- |
| Score | 1 | 0 | - |
| Colour Coded |  |  |  |

**Where: Not Clear: NC**; Not Applicable: NA.

**Quantitative quality assessment questions**

1. **Were the criteria for inclusion in the sample clearly defined?**

2	**Were the study subjects and the setting described in detail?**

3. **Was the exposure measured in a valid and reliable way?**
4. **Were objective, standard criteria used for measurement of the condition?**
5. **Were confounding factors identified?**
6. **Were strategies to deal with confounding factors stated?**
7. **Were the outcomes measured in a valid and reliable way?**
8. **Was appropriate statistical analysis used?**
